# Nitric oxide modulates the hyperalgesic response to mechanical noxious stimuli in sleep-deprived rats

**DOI:** 10.1186/1471-2202-14-92

**Published:** 2013-08-30

**Authors:** Fabio Damasceno, Gabriela O Skinner, Paulo C Araújo, Marcia MD Ferraz, Frank Tenório, Olga MMS de Almeida

**Affiliations:** 1 Department of Pharmacology and Psychobiology, Institute of Biology, State University of Rio de Janeiro, Av. 28 de Setembro, 87-Fundos, 20551-030, Rio de Janeiro, Brazil

**Keywords:** Paradoxical sleep deprivation, Nociception, NADPH-d, L-NAME

## Abstract

**Background:**

Sleep restriction alters pain perception in animals and humans, and many studies have indicated that paradoxical sleep deprivation (PSD) promotes hyperalgesia. The hyperalgesia observed after mechanical nociceptive stimulus is reversed through nitric oxide synthase (NOS) inhibition. Both nitric oxide (NO) and the dorsolateral periaqueductal gray matter (dlPAG) area of the brainstem are involved in hyperalgesia. Thus, in this work, we investigated the pain-related behavior response after mechanical noxious stimuli (electronic von Frey test), and the activity of nicotinamide adenine dinucleotide phosphate diaphorase (NADPH-d), an indicator of NOS activity, within the dlPAG of paradoxical sleep-deprived rats. We also evaluated the effects of pre-treatment with L-NAME on these parameters.

**Results:**

These data revealed that PSD reduced the hindpaw withdrawal threshold (−47%, p < 0.0001) confirming the hyperalgesic effect of this condition. In addition, there were more NADPH-d positive cells in dlPAG after PSD than in control rats (+ 59%, p < 0.0001). L-NAME treatment prevented the reduction in the hindpaw withdrawal threshold (+ 93%, p < 0.0001) and the increase in the NADPH-d positive cells number in the dlPAG of PSD-treated rats (−36%, p < 0.0001).

**Conclusion:**

These data suggest that the hyperalgesic response to mechanical noxious stimuli in paradoxical sleep-deprived rats is associated with increased NOS activity in the dlPAG, which presumably influences the descending antinociceptive pathway.

## Background

Paradoxical sleep deprivation (PSD) has been implicated in a variety of behavioral alterations [1] that are related to distinct changes in neurotransmitter systems [2]. Current research has primarily focused on the relationship between sleep and pain because periods of sleep deprivation increase pain sensitivity [3-9], and the induction of a pain-like state also interferes with sleep architecture [10]. A reduction in the analgesic effect of morphine after sleep deprivation has also been described [11,12]. Wei and colleagues demonstrated PSD-induced hyperalgesia shares common mechanisms with neuropathic pain, with the reversal of mechanical hypersensitivity through nitric oxide synthase (NOS) inhibition in paradoxical sleep-deprived and sciatic nerve-injured rats [13]. However, in a previous study, we showed that the drug amitriptylline, which is commonly used to treat neuropathic pain, did not revert/prevent PSD-induced thermal hyperalgesia, independent of the intensity of the thermal noxious stimuli or the period of sleep deprivation [14].

The periaqueductal gray matter (PAG) is a major brainstem area that plays an important role in analgesia control [15]. PAG electrical stimulation promotes analgesia in animals such as rats [16], cats [17] monkeys [18] and in humans [19]. The dorsolateral subdivision of PAG (dlPAG) exerts a critical influence on descending pain modulation [20,21].

Several studies have suggested that nitric oxide (NO) plays a role in sleep and in pain. NO is produced from L-arginine through a NOS enzyme in calcium-dependent pathways and is described as a sleep-facilitating agent [22]. During sleep deprivation, NO production increases in the basal forebrain, and both NOS inhibition and the NO scavenger prevent recovery sleep induction [22].

The effects of NO have also been described as pro– or anti–nociceptive, depending on the circumstances. Numerous studies have shown an association between NO and nociceptive signaling in chronic neuropathic pain models through upregulated NOS expression in dorsal horn neurons [23,24]. In these models, NOS inhibitors suppress induced pain [23,25]. NO immunoreactivity is associated with the nicotinamide adenine dinucleotide phosphate diaphorase (NADPH-d) enzyme, which serves as a histochemical marker for neurons that produce NO [26]. A significant increase in NADPH-d-positive neurons in PAG was observed after noxious visceral stimulation, and a decrease in these neurons was observed after acupuncture in streptozotocin-induced diabetic rats [27,28]. Some investigators have also reported that NOS inhibition augments morphine-induced analgesia in experimental animals [29].

Because the neurochemical alterations and brain sites related to the promotion of hyperalgesia in paradoxical sleep-deprived rats are not completely understood and because NOS inhibition reverses mechanical hypersensitivity in PSD [13], we hypothesized that PSD would cause a hyperalgesic effect via nitrergic neuronal changes in the dlPAG. In the present study, we examined the effect of PSD on the pain-related behavior of rats that were submitted to mechanical noxious stimuli and the expression of NOS enzyme on dlPAG, through NADPH-d histochemical analysis. We also examined these parameters in paradoxical sleep-deprived and control rats that were pre-treated with L-NAME, a nitric oxide synthase inhibitor.

## Methods

### Animal experiments

All experimental protocols followed the ethical guidelines for investigations of experimental pain in conscious animals and were previously approved by the Animal Studies Ethical Committee of the University of State of Rio de Janeiro, (CEUA/032/2010). Adult male Wistar rats (n = 44; 250–300 g) were used for all experiments. The rats were housed in cages with free access to food and water in a room under controlled light/dark cycle conditions (12 h light/12 h dark; lights on at 6:00 a.m.) and ambient temperature (23 ± 1°C).

### Paradoxical sleep deprivation

PSD was achieved using the flowerpot technique. The rats were housed individually in tanks and placed on single narrow circular platforms (6 cm diameter) surrounded by water up to 1 cm beneath the surface. The paradoxical sleep phase in the animals subjected to this procedure was completely abolished because these animals fall into the water and awake due to the muscle atonia that is characteristic of this phase. Slow wave sleep is also reduced, however does not lead to rebound sleep [30,31]. Rats from the control group were maintained in cages in the same room and for the same period of time as the experimental group.

### Paw mechanical sensitivity

Mechanical sensitivity was measured using an electronic von Frey device (Insight Equipamentos, SP, Brazil). The animals were placed in a wire chamber, where they remained still after exhibiting brief exploratory behavior. The electronic pressure transducer contacted the hindpaw through a disposable polypropylene tip. Once the rat was absolutely immobile, this propylene tip was gently pressed against the plantar surface of the hindpaw with increasing force. A single operator performed this procedure to guarantee the same strength of the delivered stimulus. Each hindpaw was tested 3 times, with an interval of approximately 15 minutes between each experimental assessment. Each single stimulus lasted no longer then 5 seconds, which was sufficient time to evoke a visible lifting of the stimulated hind limb after the unexpected touch. The corresponding force, recorded through the electronic device (in grams), was directly proportional to the length and diameter of a given classical von Frey filament. The smaller the force applied for inducing paw withdrawal, the more sensitive the animals were to the nociception stimulus.

### Tissue preparation and NADPH-d histochemical staining

The rats were deeply anesthetized (thiopental, 70 mg/kg) and transcardially perfused with 0.9% NaCl, followed by 4% paraformaldehyde fixative in 0.16 M phosphate buffer (PB) (pH 7.4) containing 10% sucrose. After perfusion, the brains were removed and post-fixed for 24 hours in a phosphate buffer solution containing 20% sucrose. The rat brains were frozen and sectioned in the coronal plane, into 60 μm sections through the dlPAG (−7.64 mm to−8.30 mm from the bregma) [32]. Four sections were collected in each well, totaling 12 coronal sections per animal. The coronal sections were stained by using 0.1 M Tris buffer (pH 8.0) containing 1 mM b-NADPH (Sigma) and 0.1 mM nitroblue tetrazolium (NBT) (Sigma) containing 0.3% Triton X-100. The sections were incubated at 37°C for 1 hour. Subsequently, the sections were washed several times in 0.1 M PB to halt the reaction, mounted on gelatinized slides and coverslipped with permount. The positive stained cells distributed throughout the dorsolateral PAG were bilaterally counted in each coronal section by an investigator blinded to the experimental condition and represent an average of 12 sections per sample. All sections were examined under light microscopy using an Olympus BX 40 microscope. Image capturing was performed with a cooled-charged-coupled device camera (Sony DXC 151A), and the images were overlaid with a fixed frame (15 μm × 15 μm) for unbiased observations.

### Study design

**Figure 1:**
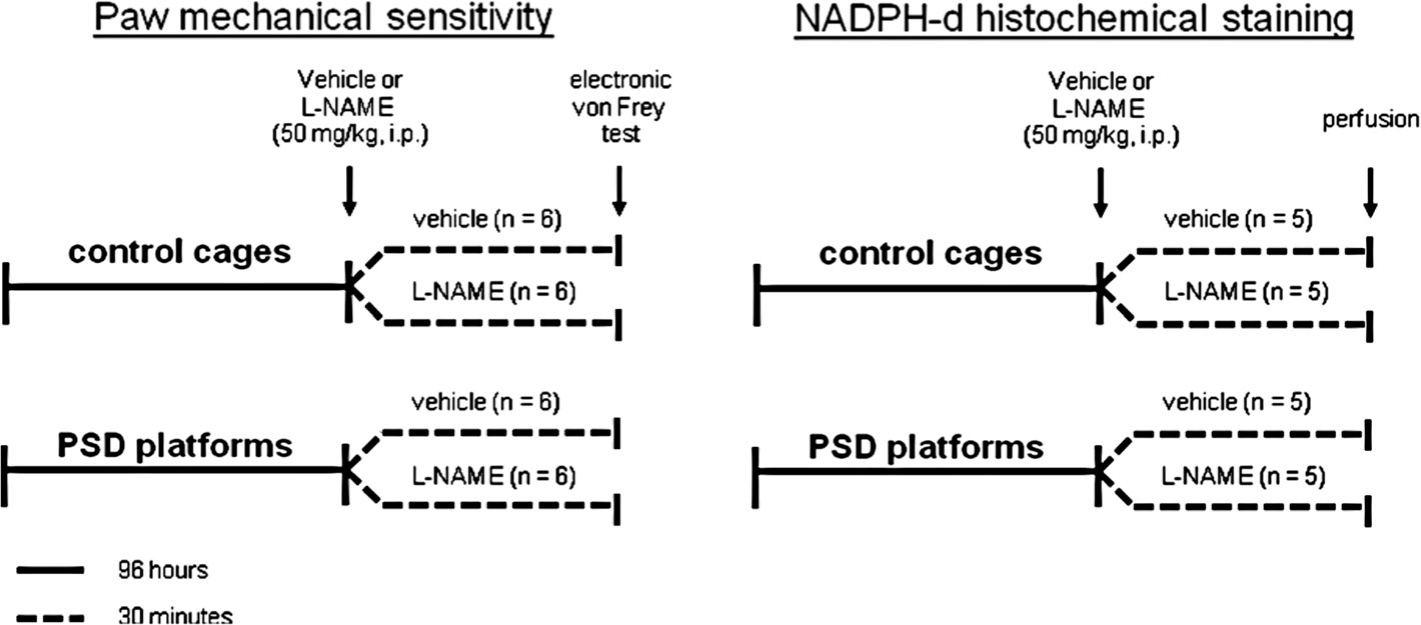
Course of study.

The animals were subjected to 96 hours of PSD (beginning and completed at 1:00 p.m.) or maintained in control cages. Subsequently, vehicle (physiological saline) or L-NAME (50 mg/kg, Sigma-Aldrich, USA) was administered (i.p.). After 30 minutes, the mechanical sensitivity was evaluated through an electronic von Frey test. The same protocol was used to perform NADPH-d histochemical staining, but instead of the electronic von Frey test, the animals were perfused for brain analysis (Figure 1).

### Statistical analysis

The data analysis was performed using GraphPad Prism 5.0 (GraphPad Software, Inc., USA), and all data are presented as the means ± S.E.M. One-way analysis of variance (ANOVA) was used to analyze the effects of L-NAME treatment on control and PSD groups subjected to the pain sensitivity mechanical test. The same statistical test was used to analyze the effects of L-NAME treatment on the number of NADPH-d positive cell bodies in the dlPAG of the control and PSD groups. When the F value was significant, the Newman–Keuls test was performed for post-hoc comparison. The results were considered significant when p ≤ 0.05.

## Results

### Mechanical nociception

**Figure 2:**
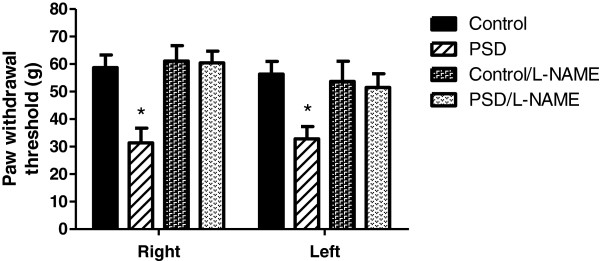
**Paw withdrawal threshold evoked using von Frey filaments in both hindpaws of the Control, PSD, and Control/L-NAME and PSD/L-NAME groups.** The data are presented as the means (g) ± S.E.M * p ≤ 0.05, different from the Control, Control/L-NAME and PSD/L-NAME groups (one-way ANOVA).

An intergroup comparison showed lower mean values for the paw withdrawal threshold of PSD-treated rats when compared to control, in both right (−47%) (F_3,92_ = 8.57, p < 0.0001) and left hindpaws (−42%) (F_3,74_ = 5.17, p < 0.0001, Figure 2). This hyperalgesic response was prevented when L-NAME was administered to PSD-treated animals. PSD/L-NAME rats presented higher mean values for the paw withdrawal threshold compared with the PSD-treated animals that received only vehicle for both the right (+ 93%) and left (+ 54%) hindpaws. There were no significant differences between the control/L-NAME and PSD/L-NAME groups for both the right (−2%) and left (−4%) hindpaws.

### NADPH-d histochemistry

**Figure 3:**
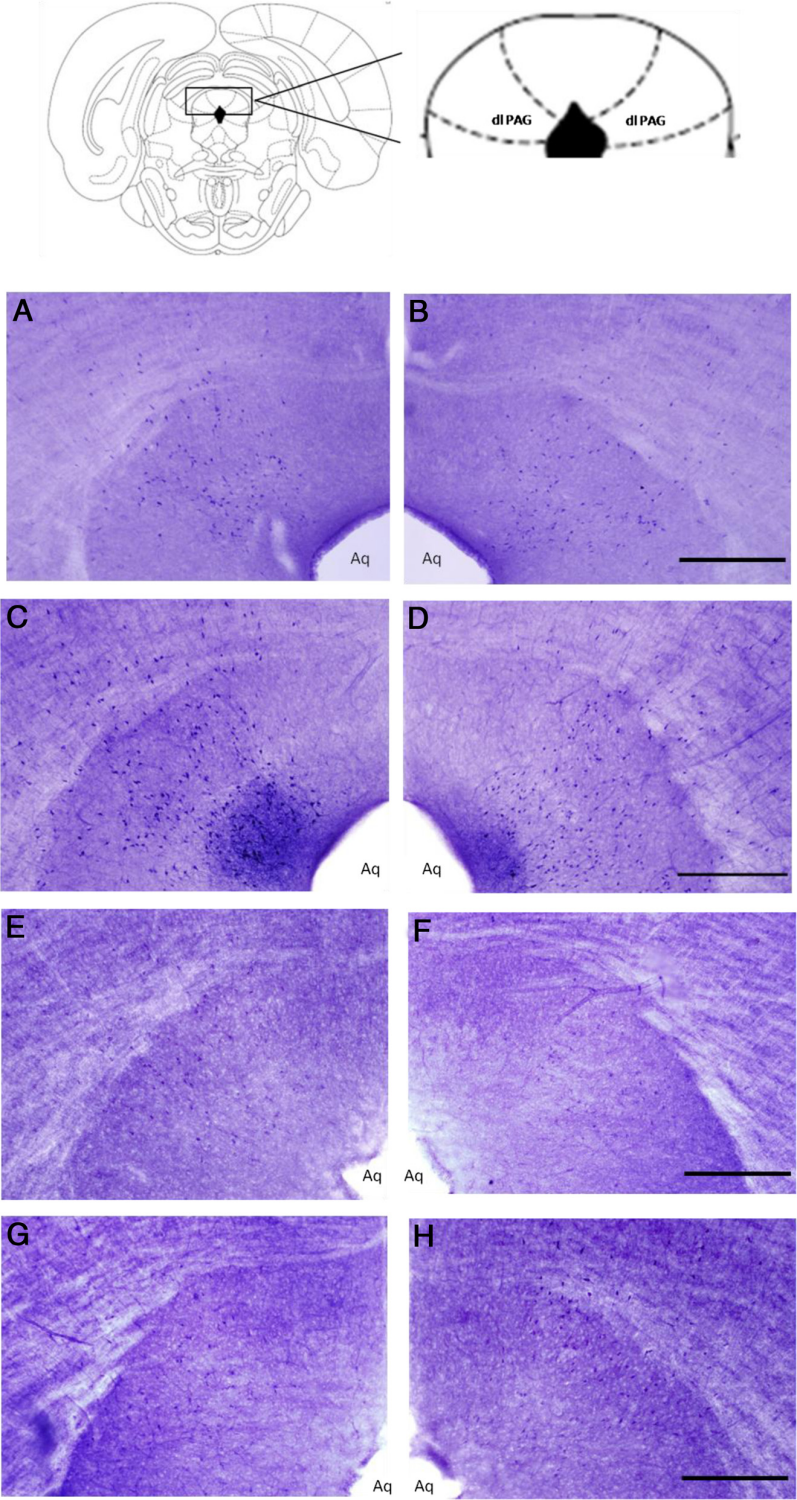
**NADPH-d-positive cell bodies in the dorsolateral periaqueductal gray matter of Control (A–left dlPAG; B–right dlPAG), PSD (C–leftdlPAG; D–right dlPAG), Control/L-NAME (E–left dlPAG; F–right dlPAG), PSD/L-NAME (G–left dlPAG; H–right dlPAG) animals.** dlPAG = dorsolateral periaqueductal gray matter; Aq = Sylvius Aqueduct; bars = 50 μm **(B**, **D**, **F**, **H)**.

**Figure 4:**
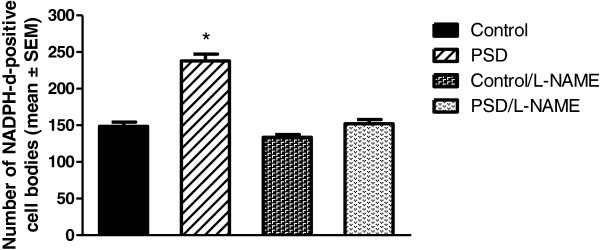
**Number of NADPH-d-positive cell bodies in the dorsolateral periaqueductal gray (dlPAG) area of the Control, PSD, Control/L-NAME and PSD/L-NAME groups.** The data are presented as the means ± S.E.M *p ≤ 0.05 different from the Control, Control/L-NAME and PSD/ L-NAME groups (one-way ANOVA).

The distribution of NADPH-d-positive cells in both control and PSD-treated rats was well defined in the two bilateral nuclei at the dorsolateral portion of the PAG (Figure 3). There was a significant increase in the NADPH-d cell number in PSD-treated rats compared with control animals (+ 60%) (F_3,207_ = 54.31, p < 0.0001). These differences were prevented when L-NAME was administered to PSD-treated animals. A comparison between groups revealed a lower mean number of NADPH-d-positive cells in the PSD/L-NAME group compared with the PSD group (–36%) (F_3,207_ = 54.31, p < 0.0001). No significant differences were observed between the control/L-NAME and PSD/L-NAME groups (+ 14%) (Figure 4).

## Discussion

In the present investigation, animals subjected to 96 hours of PSD exhibited an increased pain response after mechanical noxious stimuli, and the results suggest that there is a correlation between PSD-induced hyperalgesia and the increased NO system activity in the dorsolateral periaqueductal gray matter.

A significant reduction of the withdrawal threshold to von Frey filament application was observed after PSD. The control animals were less sensitive to pain as their paw withdrawal thresholds were higher than those of the PSD-treated rats. These data are consistent with previous studies that showed a PSD-induced hyperalgesia effect, as evaluated through mechanical noxious test [7,13]. In these studies, only one of the hindpaws was challenged with the von Frey filament. In the present study, we demonstrated that a PSD-induced hyperalgesic response to noxious mechanical stimuli is detected independently of the hindpaw selected.

An increase in NOS is associated with sleep deprivation and the development of hypersensitivity to pain in inflammatory and neuropathic pain models [22-24]. In the present study, we observed that PSD-treated animals presented an increased number of NADPH-d-positive cell bodies in the dlPAG. Because NADPH-d is a common marker for NOS and because its activity parallels NO production, this increased expression could indicate that dlPAG- NO plays a role in PSD-induced hyperalgesia. Jang and collaborators also described a NO involvement in dlPAG pain modulation [28]. These authors observed an increased dlPAG NOS activity in a peripheral neuropathy model, which was evoked through streptozotocin-induced diabetes, and a reduction after acupunctural treatment.

The observed hyperalgesia and increased NOS activity in the dlPAG were both reverted after the administration of L-NAME (50 mg/kg), a dose which was previously established as antinociceptive to mechanical, chemical and thermal noxious stimulus [33-35]. Wei and collaborators [13] also described a reduction of PSD-induced hyperalgesia through L-NAME, confirming that the nitrergic system plays an important role in this process.

In our PSD model, the increase in NADPH-d-positive cell bodies in the dlPAG might be responsible for the increased pain sensitivity because this region plays an important role in the modulation of nociception and the antinociceptive effects of morphine [20,21]. The neuronal pathways and molecular events involved in NO-supraspinal pain modulation are not completely understood. This effect is likely mediated through multiple neurobiological components. A recent study described a reversed NO-induced nociceptive hypersensitivity through the blockade of a supraspinal signaling pathway involving a PKC-dependent CREB (cAMP response element-binding protein), STAT1 (signal transducer and activator of transcription 1) and NF-κB (nuclear factor kappa B) activation in the PAG and thalamus [36]. Spinal glutamate has also been associated with mechanical hypersensitivity following sleep deprivation in rats, and the intrathecal administration of MPEP (an antagonist of mGluR5) and MK-801 (an NMDA glutamate receptor antagonist) reverts PSD-induced hyperalgesia [13]. In a previous study, we described a reduction in dopaminergic activity in the lateral PAG in paradoxical sleep-deprived rats and a reversion of hyperalgesia through L-DOPA (a dopamine precursor) treatment [3].

The PAG contains a dense plexus of cholinergic nerve terminals derived from the pontine tegmentum; these nerves mediate analgesia at least partly via the endocannabinoid signaling system [37]. The sleep-wake cycle also controls the activity of cholinergic neurons in the basal forebrain [38]. Thus, further studies are needed to characterize the neuronal pathways that are associated with sleep deprivation-induced hyperalgesia.

The PSD method used in this work induces physiologic signs of stress; however, a few studies have reported that stress produces a reduction in pain-related behavior [39,40] that is different from the PSD results obtained in this work.

Thus we propose that the hyperalgesia observed in PSD-treated rats, observed after mechanical noxious stimuli, is associated with increased NOS activity in the dlPAG and NO-signaling pathway activation, presumably influencing the descending antinociceptive pathway. Because there is a great prevalence of sleep complaints in individuals suffering from chronic pain, this knowledge will be of great pharmacological interest.

## Conclusion

Here, we present findings confirming the hyperalgesic effect observed in paradoxical sleep-deprived animals in a mechanical nociceptive behavior test. These results suggest that there is an association of PSD-induced hyperalgesia with increased NOS activity in the dlPAG.

## Abbreviations

CREB: cAMP response element-binding protein; dlPAG: Dorsolateral periaquedutal gray matter; L-DOPA: Levodopa; L-NAME: L-NG-nitroarginine methyl; mGluR5: Metabotropic glutamate receptor 5; MK801: Dizocilpine; MPEP: 2-methyl-6-(phenylethynyl)pyridine; NADPH-d: Nicotinamide adenine dinucleotide phosphate diaphorase; NF-kB: Nuclear factor kappa B; NMDA: N-methyl d-aspartate; NO: Nitric oxide; NOS: Nitric oxide synthase; PKC: Protein kinase C; PSD: Paradoxical sleep deprivation; STAT1: Signal transducer and activator of transcription 1.

## Competing interests

The authors declare that they have no competing interests.

## Authors’ contributions

FD and OM were co-principal investigators and designed the study protocol. FD, GS, PCA and MDF collected the data. FD, FT and OM analyzed and interpreted the findings and wrote the manuscript. OM performed a critical revision of the manuscript for important intellectual content and was responsible for submitting the final manuscript. All authors read and approved the final manuscript.

## References

[B1] Tufik S Andersen ML Bittencourt LR Mello MT Paradoxical sleep deprivation: neurochemical, hormonal and behavioral alterations: evidence from 30 years of research An Acad Bras Cienc 2009 81 521 538 10.1590/S0001-37652009000300016 19722021

[B2] Longordo F Kopp C Luthi A Consequences of sleep deprivation on neurotransmitter receptor expression and function Eur J Neurosci 2009 29 1810 1819 10.1111/j.1460-9568.2009.06719.x 19492440

[B3] Skinner GO Damasceno F Gomes A Almeida OM Increased pain perception and attenuated opioid antinociception in paradoxical sleep-deprived rats are associated with reduced tyrosine hydroxylase staining in the periaqueductal gray matter and are reversed by L-DOPA Pharmacol Biochem Behav 2011 99 94 99 10.1016/j.pbb.2011.04.009 21530574

[B4] Roehrs T Hyde M Blaisdell B Greenwald M Roth T Sleep loss and REM sleep loss are hyperalgesic Sleep 2006 29 145 151 16494081 10.1093/sleep/29.2.145

[B5] Lautenbacher S Kundermann B Krieg JC Sleep deprivation and pain perception Sleep Med Rev 2006 10 357 369 10.1016/j.smrv.2005.08.001 16386930

[B6] Kundermann B Krieg JC Schreiber W Lautenbacher S The effect of sleep deprivation on pain Pain Res Manag 2004 9 25 32 15007400 10.1155/2004/949187

[B7] Onen SH Alloui A Jourdan D Eschalier A Dubray C Effects of rapid eye movement (REM) sleep deprivation on pain sensitivity in the rat Brain Res 2001 900 261 267 10.1016/S0006-8993(01)02320-4 11334806

[B8] Azevedo E Manzano GM Silva A Martins R Andersen ML Tufik S The effects of total and REM sleep deprivation on laser-evoked potential threshold and pain perception Pain 2011 152 2052 2058 10.1016/j.pain.2011.04.032 21624774

[B9] Okifuji A Hare BD Do sleep disorders contribute to pain sensitivity? Curr Rheumatol Rep 2011 13 528 534 10.1007/s11926-011-0204-8 21805110

[B10] Guevara-Lopez U Ayala-Guerrero F Covarrubias-Gómez A López-Munoz FJ Torres-Gonzalez R Effect of acute gouty arthritis on sleep patterns: a preclinical study Eur J Pain 2009 13 146 153 10.1016/j.ejpain.2008.04.002 18501649

[B11] Ukponmwan OE Rupreht J Dzoljic MR REM sleep deprivation decreases the antinociceptive property of enkephalinase-inhibition, morphine and cold-water-swim Gen Pharmacol 1984 15 255 258 10.1016/0306-3623(84)90170-8 6376276

[B12] Ukponmwan OE Rupreht J Dzoljic M An analgesic effect of enkephalinase inhibition is modulated by monoamine oxidase-B and REM sleep deprivations Naunyn Schmiedebergs Arch Pharmacol 1986 332 376 379 10.1007/BF00500090 3090452

[B13] Wei H Zhao W Wang YX Pertovaara A Pain-related behavior following REM sleep deprivation in the rat: influence of peripheral nerve injury, spinal glutamatergic receptors and nitric oxide Brain Res 2007 1148 105 112 17368427 10.1016/j.brainres.2007.02.040

[B14] Damasceno F Skinner GO Gomes A Araújo PC Almeida OM Systemic amitriptyline administration does not prevent the increased thermal response induced by paradoxical sleep deprivation Pharmacol Biochem Behav 2009 94 51 55 10.1016/j.pbb.2009.07.005 19619573

[B15] Heinricher MM Tavares I Leith JL Lumb BM Descending control of nociception: specificity, recruitment and plasticity Brain Res Rev 2009 60 214 225 10.1016/j.brainresrev.2008.12.009 19146877 PMC2894733

[B16] Murotani T Ishizuka T Nakazawa H Wang X Mori K Sasaki K Ishida T Yamatodani A Possible involvement of histamine, dopamine, and noradrenalin in the periaqueductal gray in electroacupuncture pain relief Brain Res 2010 1306 62 68 19819232 10.1016/j.brainres.2009.09.117

[B17] Horie H Pamplin PJ Yokota T Inhibition of nociceptive neurons in the shell region of nucleus ventralis posterolateralis following conditioning stimulation of the periaqueductal grey of the cat: evidence for an ascending inhibitory pathway Brain Res 1991 561 34 42 1797348 10.1016/0006-8993(91)90746-i

[B18] Willis WD Gerhart KD Willcockson WS Yezierski RP Wilcox TK Cargill CL Primate raphe-and reticulospinal neurons: effects of stimulation in periaqueductal gray or VPLc thalamic nucleus J Neurophysiol 1984 51 467 480 6422009 10.1152/jn.1984.51.3.467

[B19] Richardson DE Akil H Pain reduction by electrical brain stimulation in man Part 1: acute administration in periaqueductal and periventricular sites J Neurosurg 1977 47 178 183 10.3171/jns.1977.47.2.0178 327030

[B20] Suplita RL II Farthing JN Gutierrez T Hohmann AG Inhibition of fatty-acid amide hydrolase enhances cannabinoid stress-induced analgesia: sites of action in the dorsolateral periaqueductal gray and rostral ventromedial medulla Neuropharmacology 2005 49 1201 1209 10.1016/j.neuropharm.2005.07.007 16129456

[B21] Walker JM Huang SM Strangman NM Tsou K Sañudo-Peña MC Pain modulation by release of the endogenous cannabinoid anandamide Proc Natl Acad Sci 1999 96 12198 12203 10.1073/pnas.96.21.12198 10518599 PMC18435

[B22] Kalinchuk AV Stenberg D Rosenberg PA Porkka-Heiskanen T Inducible and neuronal nitric oxide synthases (NOS) have complementary roles in recovery sleep induction Eur J Neurosci 2006 24 1443 1456 10.1111/j.1460-9568.2006.05019.x 16987226

[B23] Miclescu A Gordh T Nitric oxide and pain: ‘something old, something new’ Acta Anesthesiol Scand 2009 53 1107 1120 10.1111/j.1399-6576.2009.02054.x 19702699

[B24] Lam HH Hanley DF Trapp BD Saito S Raja S Dawson TM Yamaguchi H Induction of spinal cord neuronal nitric oxide synthase (NOS) after formalin injection in the rat hind paw Neurosci Lett 1996 210 201 204 10.1016/0304-3940(96)12702-6 8805130

[B25] Chapman V Buritova J Honoré P Besson JM 7-Nitro-indazole, a selective inhibitor of neuronal nitric oxide synthase, reduces formalin evoked c-Fos expression in dorsal horn neurons of the rat spinal cord Brain Res 1995 697 258 261 10.1016/0006-8993(95)00973-T 8593586

[B26] Hope BT Michael GJ Knigge KM Vincent SR Neuronal NADPH diaphorase is a nitric oxide synthase Proc Natl Acad Sci 1991 88 2811 2814 10.1073/pnas.88.7.2811 1707173 PMC51329

[B27] Rodella L Rezzani R Agostini C Bianchi R Induction of NADPH-diaphorase activity in the rat periaqueductal gray matter after nociceptive visceral stimulation Brain Res 1998 793 333 336 10.1016/S0006-8993(98)00255-8 9630710

[B28] Jang MH Shin MC Koo GS Lee CY Kim EH Kim CJ Acupuncture decreases nitric oxide synthase expression in periaqueductal gray area of rats with streptozotocin-induced diabetes Neurosci Lett 2003 337 155 158 10.1016/S0304-3940(02)01318-6 12536047

[B29] Toda N Kishioka S Hatano Y Toda H Modulation of opioid actions by nitric oxide signaling Anesthesiology 2009 110 166 181 10.1097/ALN.0b013e31819146a9 19104184

[B30] Machado RB Hipólide DC Benedito-Silva AA Tufik S Sleep deprivation induced by the modified platform technique: quantification of sleep loss and recovery Brain Res 2004 1004 45 51 10.1016/j.brainres.2004.01.019 15033418

[B31] Maloney KJ Mainville L Jones BE Differential c-Fos expression in cholinergic, monoaminergic, and GABAergic cell groups of the pontomesencephalic tegmentum after paradoxical sleep deprivation and recovery J Neurosci 1999 19 3057 3072 10191323 10.1523/JNEUROSCI.19-08-03057.1999 PMC6782283

[B32] Paxinos G Watson C The rat brain in stereotaxic coordinates 1982 Sydney: Academic Press 10.1016/0165-0270(80)90021-7 6110810

[B33] Handy RLC Moore PK Effects of selective inhibitors of neuronal nitric oxide synthase on carrageenan-induced mechanical and thermal hyperalgesia Neuropharmacology 1998 37 37 43 10.1016/S0028-3908(97)00201-3 9680257

[B34] Tassorelli C Greco R Wang D Sandrini G Nappi G Prostaglandins, glutamate and nitric oxide synthase mediate nitroglycerin-induced hyperalgesia in the formalin test Eur J Pharmacol 2006 534 103 107 10.1016/j.ejphar.2006.01.023 16507304

[B35] Chen Y Boettger MK Reif A Schmitt A Üçeyler N Sommer C Nitric oxide synthase modulates CFA-induced thermal hyperalgesia through cytokine regulation in mice Mol Pain 2010 6 13 10.1186/1744-8069-6-13 20193086 PMC2838835

[B36] Galeotti N Ghelardini C Reversal of NO-induced nociceptive hypersensitivity by St. John’s wort and hypericin: NF-κB, CREB and STAT1 as molecular targets Psychopharmacology (Berl) 2013 227 149 163 10.1007/s00213-012-2950-3 23254377

[B37] Lau BK Vaughan CW Muscarinic modulation of synaptic transmission via endocannabinoid signalling in the rat midbrain periaqueductal gray Mol Pharmacol 2008 74 1392 1398 10.1124/mol.108.045872 18678620

[B38] Modirrousta M Mainville L Jones BE Dynamic changes in GABAA receptors on basal forebrain cholinergic neurons following sleep deprivation and recovery BMC Neurosci 2007 8 15 10.1186/1471-2202-8-15 17316437 PMC1805759

[B39] Jimenez-Velazquez G Fernandez-Guasti A Lopez-Muñoz FJ Influence of pharmacologically-induced experimental anxiety on nociception and antinociception in rats Eur J Pharmacol 2006 547 83 91 10.1016/j.ejphar.2006.06.060 16952350

[B40] Butler RK Finn DP Stress-induced analgesia Progr Neurobiol 2009 88 184 202 10.1016/j.pneurobio.2009.04.003 19393288

